# Assessing the welfare of dogs surgically sterilized during single day free community pet clinics in Kampala Metropolitan area, Central Uganda

**DOI:** 10.1017/awf.2026.10071

**Published:** 2026-02-18

**Authors:** Zozianne Hoareau, Lewis Ashabahebwa, William Lume, Kelvin Bwambale, Suzanne T. Millman, Dickson Stuart Tayebwa

**Affiliations:** 1 Department of Veterinary Pharmacy and Clinical Studies, Makerere University, Kampala, Uganda; 2 Director, Jinja Institute of Technology, Uganda; 3 Department of Biostatistics and Epidemiology, College of Health Sciences, Makerere University, Kampala, Uganda; 4 Department of Veterinary Diagnostic and Production Animal Medicine, Iowa State University, Ames, United States

**Keywords:** Animal welfare, community clinics, dog population management, pre-surgical evaluation, spay-neuter, Uganda

## Abstract

Free community pet clinics (FCPCs) are instrumental in providing healthcare services for pets in resource-constrained communities. These programmes are typically single-day and coupled with limited opportunity for post-operative follow-up, the likelihood of compromise on the welfare of the sterilised pets becomes apparent. The objective of this study was to identify welfare and ethical challenges associated with dog sterilisation clinics conducted in Kampala Metropolitan Area, Uganda, from May to October 2023. We evaluated the welfare of 46 dogs sterilised at the FCPCs. We collected data using a questionnaire combined with observations and checklists at all stages, including arrival, pre-surgical evaluation, post-surgical recovery, and follow-ups at 7 and 30 days post-surgery. Dogs transported to the clinics by motorcycle moved the longest distance (9.5 km; range: 7.2–11 km), followed by those brought by car (3.8 km; range 3.8–4 km) and lastly by walking (2.0 km; 1–3 km). During the pre-surgical waiting period and post-surgical recovery, 91.3 and 63.0% of dogs, respectively, did not receive any provisions. The Animal Welfare Assessment Grid (AWAG) tool evaluated the impact of physical, psychological, and procedural factors on dog welfare and revealed that procedural events had the most negative impact. In the follow-up, one week post-surgery, a significant percentage of dogs were healing normally (73.7%) while infection at the surgical site was reported in 15.4%. Assessing the behavioural change of dogs by 30 days post-operatively, 46.4% reportedly had increased aggression levels. These findings highlight crucial welfare and stress control points for dogs sterilised at free community clinics, which may influence the outcome of the surgery and later interactions with healthcare professionals.

## Introduction

Free-roaming dogs constitute approximately 75% of the estimated 700 million dog population worldwide (Smith *et* *al.* 2019). Although the exact number of stray dogs in Uganda remains unknown, estimates put it at around 1.4 million (Tayebwa *et* *al.* 2024b), out of the 2.1 million registered nationwide (UBOS 2021). The high prevalence of free-roaming dogs in Uganda is largely attributed to irresponsible pet ownership practices, such as low utilisation of breeding control practices and inability to confine dogs (Tayebwa *et* *al.* 2024b). The Kampala Metropolitan Area (KMA) is comprised of densely populated urban centres, providing a niche for roaming dogs due to the availability of hiding places, such as abandoned buildings (Reese & Vertalka 2021), and availability of food from garbage piles (Wright *et* *al.* 2021).

Traditionally, culling has been the primary roaming dog population control method used by low-middle income countries, typically by poisoning (Dalla Villa *et* *al.* 2010). However, in 1990, the World Health Organisation discouraged the use of culling and recommended alternative methods, such as registration and identification, vaccination, public education, and surgical sterilization (Bögel *et* *al.* 1990). Surgical sterilisation gained particular popularity due to the immediacy and permanency of its prevention of further breeding at individual level, and mainly took the form of high-volume, stationary, non-profit, spay-neuter clinics (White *et al.* 2018).

Canine surgical sterilisation entails procedures such as ovariohysterectomy for females and orchiectomy for males, aimed at making dogs incapable of reproduction (Dugassa *et al.* 2020; Kutzler 2020). By eliminating the ability to reproduce, these procedures reduce the number of unwanted litters, essentially halting the rise in unwanted free-roaming dog populations, provided the death/removal rate is higher than the replacement rate. This plays a crucial public health role by lowering the transmission of zoonotic diseases and minimising incidents of dog bites (Stull *et al.* 2015; Duncan-Sutherland *et al.* 2022).

In a clinic/hospital setting, canine surgical sterilisation procedures are conducted in controlled environments with access to advanced medical equipment and trained technical staff (Romagnoli *et* *al.* 2024). Despite the advantages of this setting, it is cost-prohibitive and inaccessible to pet owners in rural or underserved areas where access to veterinary clinics or hospital services may be limited (Åsbjer 2009; White *et al.* 2018). Instead, mobile clinics or temporary set-ups have been used as alternatives to provide access to veterinary services in rural areas (Makolinski 2012). In Uganda, temporary set-ups in the form of free community pet clinics (FCPCs) are made possible through public-private partnerships (Isiko *et* *al.* 2017). Non-governmental organisations, such as the Uganda Society for the Protection and Care of Animals (USPCA), the Vetconekt Initiative, Uniquely Paws, the Big Fix Uganda among others collaborate with government agencies to deliver essential pet healthcare services. These include rabies vaccinations, ecto- and endo-parasite control, grooming, and surgical sterilisation (Ali & Zeidan 2001; Evans *et al.* 2019).

Free sterilisation programmes offer a humane solution for controlling free-roaming dog populations, replacing the once-common practice of mass dog poisoning, which was widely used in Uganda (Alobo *et al.* 2020). Although FCPCs offer numerous advantages and are gaining increasing interest amongst resource-limited communities, concerns remain regarding the welfare of the sterilised dogs (Bacon *et al.* 2017). There is a need to evaluate the suitability of dogs subjected to surgery, especially when owners eager to take advantage of the opportunity may overlook potential health risks. Additionally, the lack of post-operative care is a significant concern, as these clinics are often single day events with limited opportunities for patient follow-up (Bacon *et al.* 2017). Assessing the welfare of dogs during these events can offer valuable insights and highlight areas for improvement, ensuring that FCPCs do not compromise the well-being of the pets sterilized (Bacon *et* *al.* 2017). In this study, we examined the potential welfare challenges faced by dogs sterilised at FCPCs in the KMA. Additionally, we evaluated the provisions given and also monitored post-operative complications. Our findings offer a critical insight and suggest key improvements to enhance the welfare of dogs during FCPS held in KMA.

## Materials and methods

### Ethical approval

This study protocol was approved by the School of Veterinary Medicine and Animal Resources Institutional Animal Care and Use Committee (No SVAR_IACUC/150/2023). Informed consent was obtained from the owners of dogs included in this study.

### Area of study

**Figure 1.**
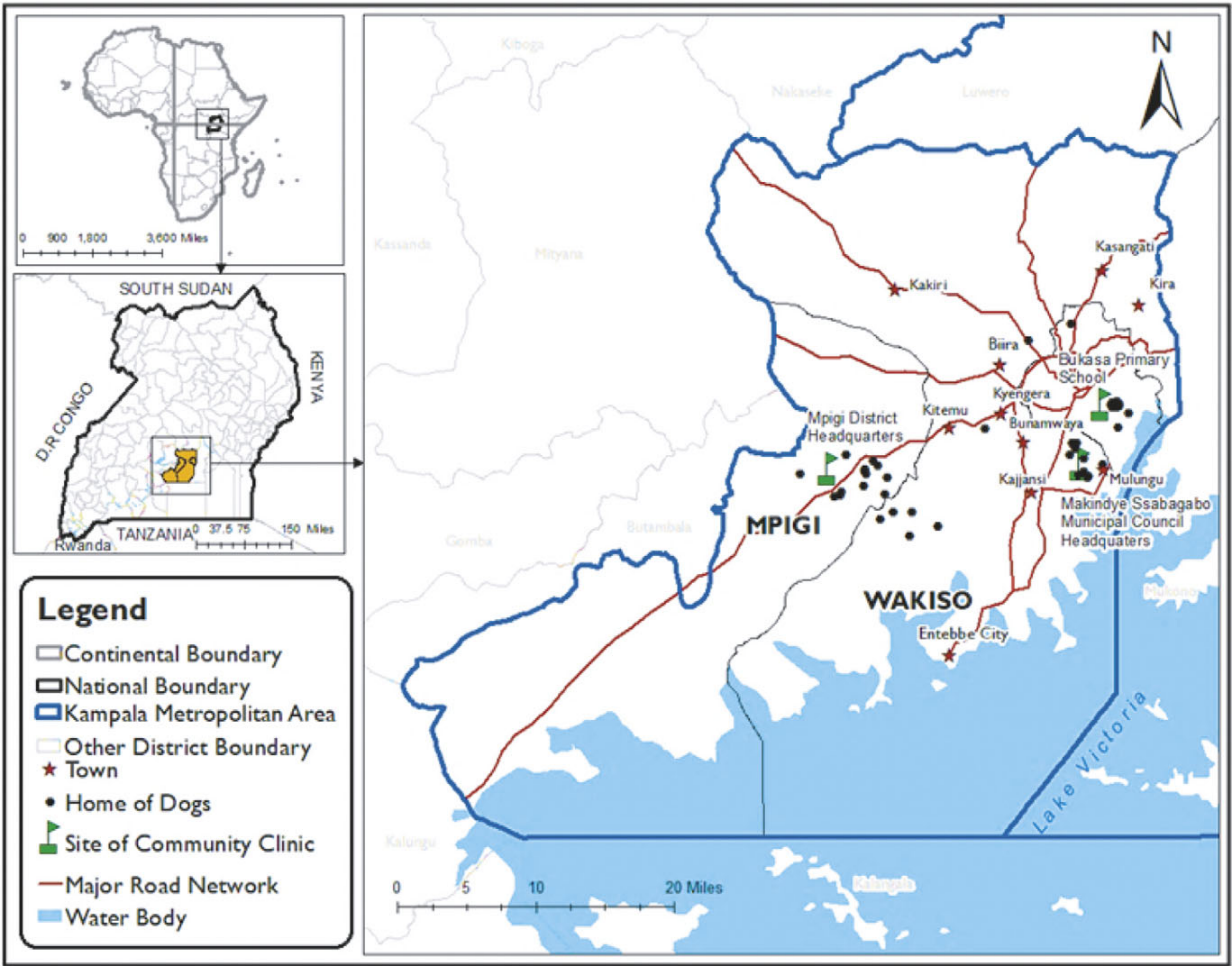
Map showing the locations of the homes (round dots) of the dogs (n = 46) brought to the single day free community pet clinics in Kampala Metropolitan area, Central Uganda for neutering and the locations of the clinics (flag symbol) where data were collected.

Research took place within the Kampala Metropolitan Area (KMA) which encompasses the capital city and its surrounding districts, including, Wakiso, Mukono and Mpigi (Figure 1). The selection of this area was predicated on the fact that KMA includes trading centres and slum settings (Ismail 2020). These conditions allow for a high concentration of roaming dogs amidst an ever-growing human presence, an amalgam that has resulted in dog attacks on both livestock and humans (Tayebwa *et al.* 2024a). Furthermore, the USPCA, Uganda’s most notable animal shelter that organises these FCPCs, is located in Kampala city. Therefore, the majority of the spay and neuter programmes tend to be organised in Kampala and its surrounding areas.

### Study design

We used a longitudinal study approach with questionnaires, observations, and calculations to gain insights into the welfare of surgically sterilised dogs at FCPCs. We assessed the mode of transportation to and from the clinic site, handling, and care of the dogs, the waiting time before surgery, recovery time after surgery, wound healing, and changes in behaviour after the surgical procedure.

### Enrolment and sample size estimation

We used a convenience sampling method of clients attending three FCPCs. Owners who did not wish for their dog to be sterilised were provided with other veterinary services offered by the FCPC but were excluded from the study. Clinicians determined whether the dog was a surgery candidate based upon the pre-surgery assessment, excluding those that were of poor surgical risk status.

### Data collection

To reduce inter-assessor variability, the three assessors from the research team, who were veterinary professionals, underwent training in data collection at a preliminary FCPC to familiarise themselves with the protocol. Although a formal statistical analysis of inter-rater reliability was not performed, consistency was monitored throughout data collection. The research team conducted periodic group reviews of randomly selected assessments to ensure alignment, maintaining a minimum agreement level of two out of three assessors for each item. The principal investigator was the referent against whom data were compared, i.e. if two assessors disagreed, the third assessor resolved the discrepancy.

### Assessing the mode of transportation to and from the clinic and distance covered

Informed consent was obtained from owners upon their arrival at the clinic. We used a questionnaire and observation to collect information regarding the means of transport used and the location of the homes of the dogs. Global Positioning System (GPS) coordinates of the clinic site and those of the homes of the dogs were determined using Google maps and used to estimate the distance covered by the owners and their dog.

### Assessing the conditions of the dog during the waiting period prior to surgery

Periods between key events (i.e. from arrival at the clinic to induction of anaesthesia, and from recovery from anaesthesia to departure from the clinic) were timed using a stopwatch for each dog and any provisions afforded by the FCPC organisers were recorded using a structured checklist.

**Provisions:** Prior to surgery, we were specifically interested in provision of drinking water and shade from direct sunlight for the dog. After the surgery, we were interested in provision of soft bedding for the dog to lie upon as well as covering with a blanket to offset the threat of hypothermia.

**Animal Welfare Assessment Grid:** A slightly modified version of the Animal Welfare Assessment Grid (AWAG) was employed to assess the welfare of the dogs at the clinic. This tool was previously utilised by Malkani *et al.* (2022). The system uses four parameters to measure the impact on welfare: physical health; psychological well-being; environmental comfort; and veterinary and managemental procedural events. However, we excluded the environmental section since it focused on factors relating to the home environment rather than that at the FCPCs. Each parameter was subdivided into several factors that contributed to the overall welfare score. These various factors were scored between one and ten. Each factor score was defined using descriptors for each number to reduce scoring bias. Therefore, a lower score indicated a lower possible impact on welfare, whilst higher scores indicated higher possible impact on welfare for each respective factor. For each parameter, mean factor scores were then calculated to ascertain what parameters were impacting welfare at that point in time (for additional information, see Table S1 in section 1 of the Supplementary material).

**Pre-surgical examination:** We also conducted a pre-surgical examination of all dogs to be sterilised to determine surgical suitability by adopting some of the parameters from the clinical evaluation form provided in the IFAW (2017) field guidelines for spay-neuter campaigns. We specifically investigated rectal temperature, body condition score and hydration status. These parameters were selected based upon practicality, relevance to immediate health and welfare, and applicability under the time and resource constraints of the community clinic setting. We also screened for ectoparasites (ticks and fleas) because they are geographically endemic, are a well-known problem among roaming dogs in Uganda (Hyeroba *et al.* 2017), and they vector pathogens that cause diseases/conditions which could adversely affect the outcome of surgical procedures (Jacobson *et al.* 2000, Hattersley *et al.* 2020).

Upon arrival at the clinic dogs were afforded a minimum of 10 min rest by organisers, either sitting or lying down in an open unshaded area to allow dogs to acclimate after travel stressors. Rectal temperature was measured using a digital thermometer (Control D® CDT01) inserted per rectum with the metallic tip positioned in contact with the rectal mucosa. Temperatures ranging between 37.5 and 39. 2°C were considered as being within the normal range (Reece *et al.* 2015). For dogs showing an elevated temperature, fever was ruled out by measuring it in triplicates at 5-min intervals. If the temperature continued to rise, or dropped but stayed above normal by the final measurement, the animal was considered hyperthermic as shown by Carter *et al.* (2024). However, if readings remained relatively unchanged, the dog was considered febrile since a higher hypothalamic temperature set-point is achieved and maintained by the body’s thermoregulatory mechanisms (Nakamura 2024).

We scored body condition using a 9-point scale adapted from Petrean *et al.* (2023): 1/9 emaciated; 2/9 very thin; 3/9 thin; 4–5/9 ideal weight; 6/9 overweight; 7/9 heavy; 8/9 obese; and 9/9 severely obese.

Hydration was assessed using skin turgor as described previously by Goucher *et al.* (2019). A thumb and forefinger was used to elevate a fold of skin parallel to the sagittal crest on the parietal bone to a height approximating 2 cm for roughly 3 s. The skin fold was released and the time taken for the fold to return to normal was noted. The score was interpreted as normal/adequate if the fold collapsed within 1 s or less, marginal if within 2–3 s and inadequate if it took 3 s or more.

Ectoparasite presence was assessed visually by a thorough physical inspection of the skin and fur as described by Smith *et al.* (2011). We brushed our fingers through the dog’s hair from head to tail and from tail to head, applying sufficient pressure to palpate any ticks; paying special attention to ears, neck and chest areas, legs, axillae and between the toes. Finally, we parted the hair down the length of the body, exposing the skin underneath to check visually for fleas. Any parasite observed was recorded as; fleas only, ticks only, both fleas and ticks or none.

An overall physical fitness score was then determined from body condition score, rectal temperature, hydration status and ectoparasite presence findings, which were scored as excellent, good, fair, poor and critical according to the RAVS animal condition score. For instance, an animal was scored as excellent if there were no significant care-related abnormalities on physical examination, ideal body condition, no indication of external parasite infestations and potential minor systemic conditions that are not a result of problems with client care/attention (for additional information see section 2; Supplementary material).

**Anaesthetic protocol:** We reported the anaesthetic protocol used at the clinics due to its significant effects on behavioural modulations seen in dogs after surgery (Fox *et* *al.* 2000). N.B. This was a pre-existing protocol and we were not responsible for its development. Antibiotic therapy and pain medication administered were also reported.

### Monitoring canine welfare post-surgery

**Post-surgery prior to recovery from anaesthesia**: We used a checklist to assess the wound for any swelling or discharge and the dogs’ recovery process as previously described (Fossum *et* *al.* 2018).

**Day 7 post-surgery:** We followed up on Day 7 and examined the wounds to determine apposition, discharge, swelling and dehiscence. The inflammatory phase usually spans the first 1 to 5 days after surgery, followed by the proliferative phase which typically spans from day 5 to day 21 after surgery. This phase is characterised by robust tissue regeneration and the formation of essential structures that contribute to the healing process (Grubbs & Manna 2023).

**Day 30 post-surgery:** All the owners of animals that had participated in the study were contacted via telephone in order to assess long-term behavioural modifications following the sterilisation surgery, in particular aggression towards humans or other animals, given the sheer range of literature that exists describing the varied effects sterilisation can have on aggressive behaviour in dogs (Farhoody *et al.* 2018; Pipan & Pavlin 2023; Hess *et al.* 2024).

The questions asked were:

- How does your dog typically behave around other dogs?
- Have you observed any changes in how your dog interacts with other dogs since the surgery? If yes, please describe.
- How does your dog behave towards people they are familiar with?
- How does your dog behave towards people they are unfamiliar with?
- Has this behaviour changed in any way since sterilisation?

Any responses mentioning aggressive behaviour such as growling, snarling, barking, and lunging towards humans and/or other dogs were recorded, specifically instances in which the dog had become: less aggressive; more aggressive; or not changed behaviour since the surgery.

### Statistical analysis

Data from Microsoft Excel® 2016 was imported into STATA version 14.2 for analysis. For univariate analysis, mean and standard deviations were obtained for continuous variables, such as distance to the community clinic (in km) and time (in min), while for categorical variables, such as provisions (absent/present), hydration status (adequate/marginal/inadequate), frequencies and percentages were reported.

For bivariate analysis, the Kruskal-Wallis test was used to compare the median distance (in km), a continuous variable, across different categories of the mode of transport (motorcycle/walking/car), since the approximate distance in km was not normally distributed and did not meet the assumptions for one-way ANOVA. *Post hoc* pairwise comparisons were conducted using Dunn’s test to identify specific group differences. Relationships between categorical variables were assessed using the Fisher’s exact test, while those between continuous variables were checked with linear regression. Two-way ANOVA was used to test the influence of various independent factors like transport type and ambient temperature on core body temperature. Significance was determined at alpha of 5% thus *P*-values ≤ 0.05 were reported as significant.

## Results

### Demographic data for dogs surgically sterilised

A total of 46 dogs were surgically sterilised at the three sites located in Kampala, Wakiso and Mpigi districts. Out of the potential 30 that were brought to the clinic, 21 (70%) dogs were enrolled in the study in the first FCPCs. At the second, 12 out of 13 (92%) possible dogs were enrolled, while 13 out of 20 (65%) were enrolled at the final FCPCs. Of the dogs sterilised, 24 (52.2%) were male and 22 (47.8%) were female. All dogs were local breeds (see Figure 3). Regarding the age, 25 (54%) were adults, 20 (43%) were puppies, and 1 (2%) was geriatric.

### Mode of transportation and average distance travelled

**Figure 2.**
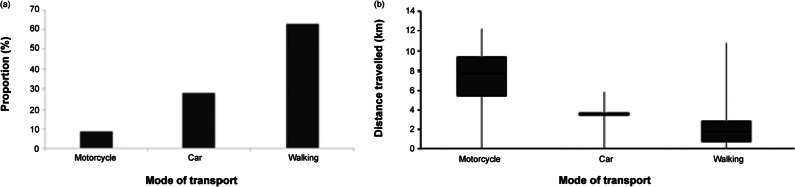
Bar graphs showing (a) proportion of dogs (n = 46) arriving at the free community pet clinics in Kampala Metropolitan area, Central Uganda relative to mode of transport and (b) median distances travelled using the different modes of transportation.

Transport to the FCPC is depicted in Figure 2. The average ambient temperature on clinic days was 25°C (range: 24–26°C) while average relative humidity was 65.7% (range: 62–69%). Most of the dogs (63.0%) came to the site on foot, followed by those who were transported by a privately owned car (28.3.%). The most seldom used mode of transportation was public motorcycle, known locally as a *boda-boda* (8.7%). The median distance traveled was highest using *boda-boda* transportation (9.5 km; range: 7.2–11 km), while cars covered 3.8 km (range: 3.8–4 km), and those who walked covered 2.0 km (1–3 km). The Kruskal-Wallis test revealed a significant difference in the median approximate distance traveled (*P* = 0.0189) among the three modes of transport. *Post hoc* Dunn’s test showed that the significant differences were between *boda-boda* and walking (*P* = 0.0100) and car and walking (*P* = 0.0222), while the difference between *boda-boda* and car did not differ significantly (*P* = 0.1596).

**Figure 3.**
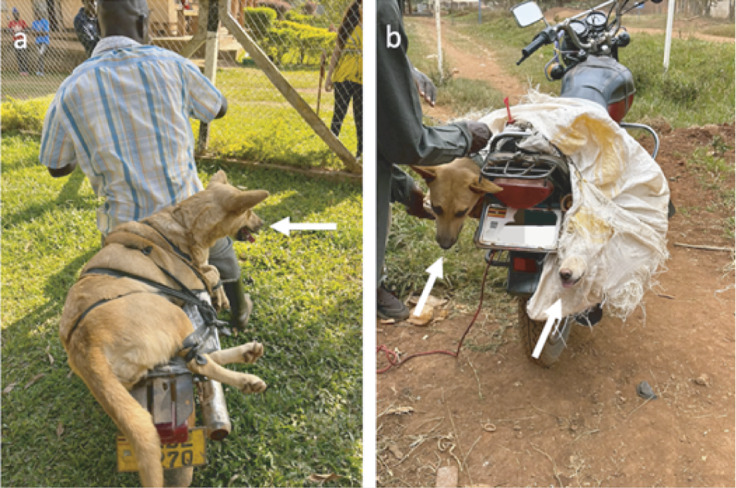
The images show dogs being transported on public motorcycles, locally known as *boda-bodas.* For (a) a dog is secured to carrier with a rubber strap while (b) depicts two dogs in a sisal sack, also fastened to the motorcycle.

To transport dogs by motorcycle, owners typically tied their dogs to the motorbike seat carrier using a rope or elastic belt (Figure 3[a]), placed them in a sack (Figure 3[b]), or carried them on their laps.

### Use of the Animal Welfare Assessment Grid

**Figure 4.**
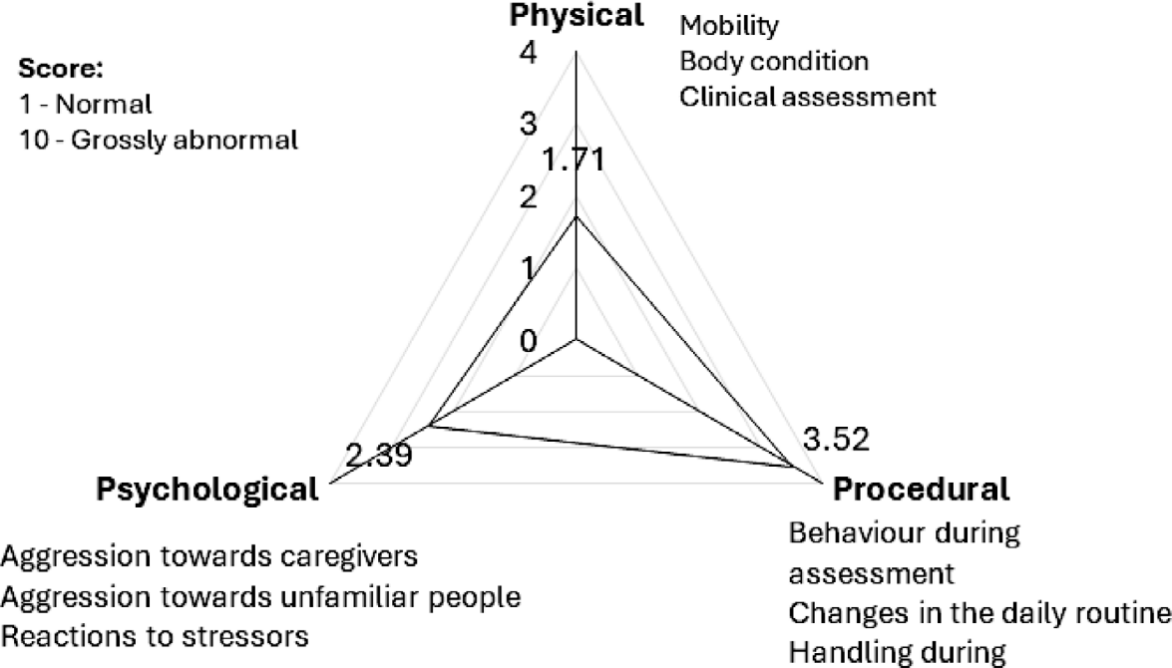
Radar chart representing the animal welfare assessment grid score for dogs (n = 46) arriving at free community pet clinics in Kampala Metropolitan area, Central Uganda for neutering, showing the average scores across physical, procedural, and psychological domains, with specific sub-parameters listed for each, and a scoring system ranging from 1 (Normal) to 10 (Grossly abnormal).

Procedural events had the most substantial negative impact on welfare, averaging a score of 3.52. This was then followed by psychological factors with a score of 2.39. Physical factors had the most significant positive impact on the welfare of the dog with 1.71 (see Figure 4).

### Provisions given by the organisers

**Figure 5.**
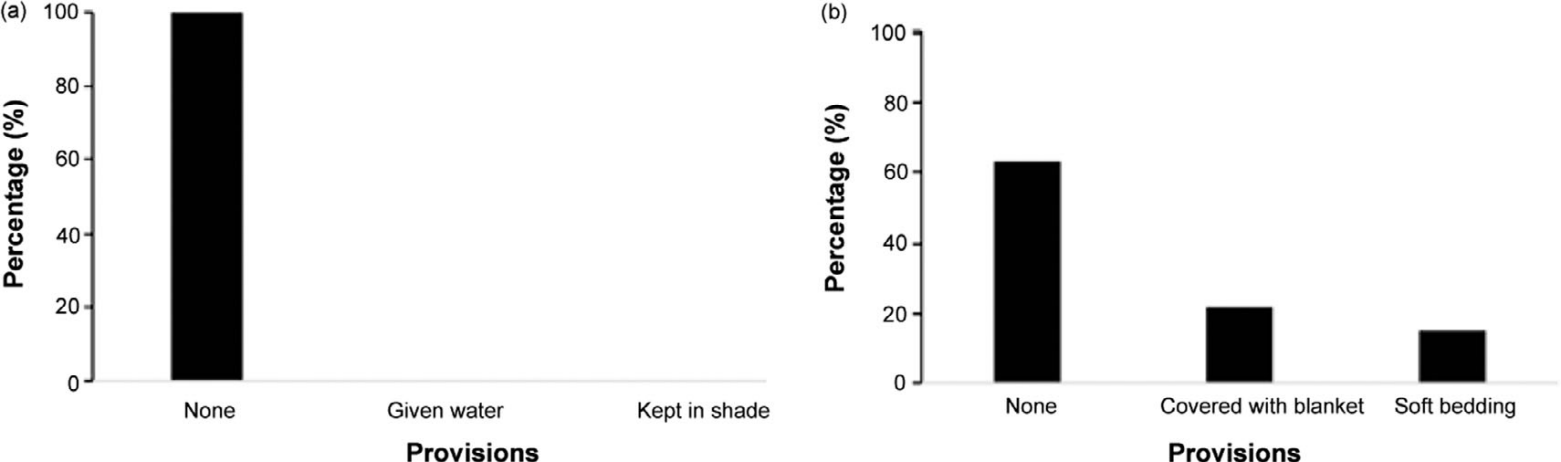
Bar graphs showing provisions received by dogs enrolled in the study (n = 46) arriving at free community pet clinics in Kampala Metropolitan area, Central Uganda (a) before surgery and (b) after surgery.

Dogs spent a mean (± SD) time of 201.7 (± 144.8) min at the clinic. The process from arrival to anaesthetic induction took 151.4 (± 100.1) min. After surgery, it took 50.3 (± 44.7) min for the dogs to recover and leave the clinic. Prior to surgery, none of the dogs received provisions. Post surgery, soft bedding and blankets were accessed by 15.2 and 21.8% of the dogs, respectively (see Figure 5).

### Pre-surgical examination findings

**Figure 6.**
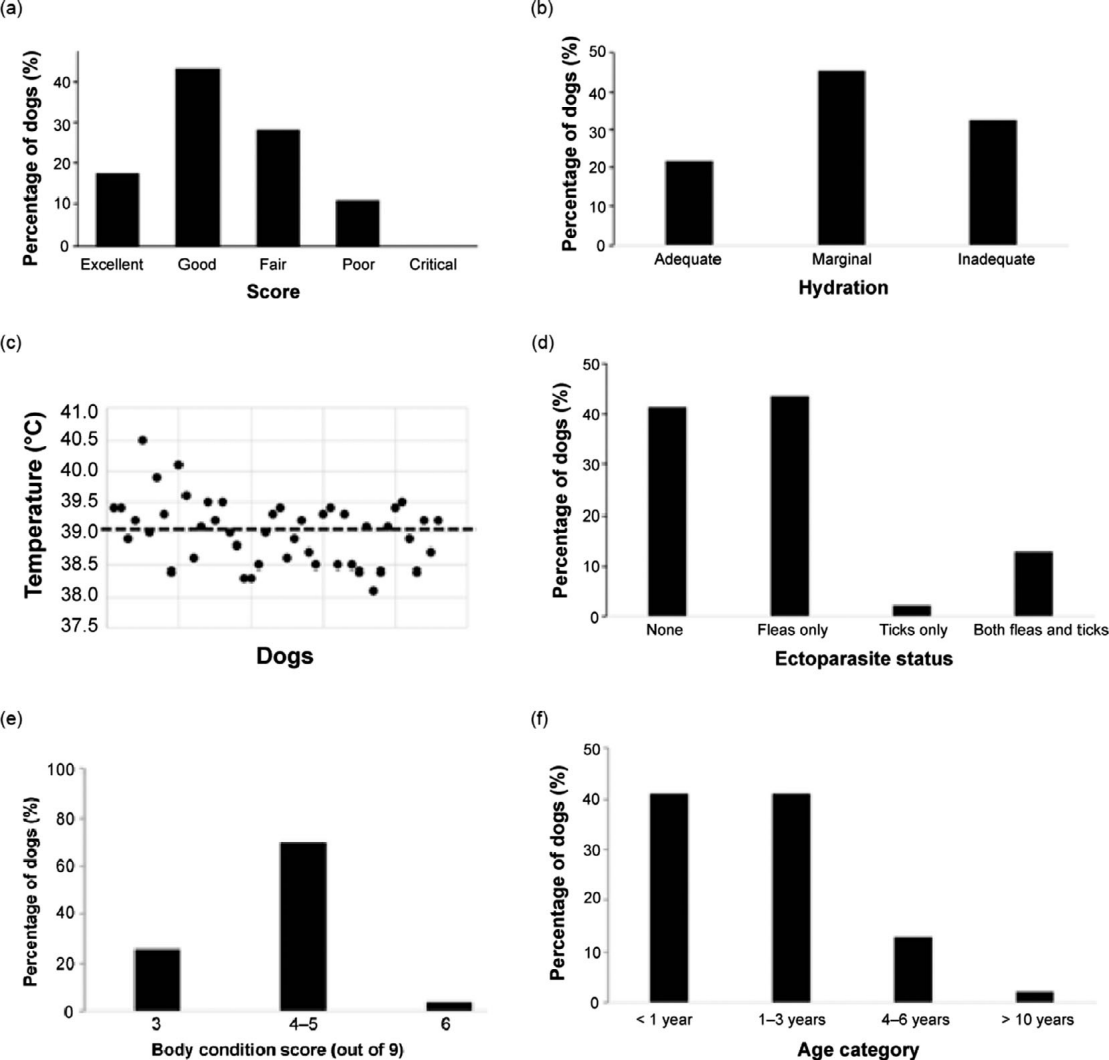
Pre-surgical examination findings of the dogs (n = 46) enrolled in the study arriving at free community pet clinics in Kampala Metropolitan area, Central Uganda showing (a) overall fitness score, (b) hydration status, (c) temperature, (d) ectoparasite presence, (e) body condition score (Petrean *et* *al.* 2023) and (f) age category.

As shown in Figure 6, 39% (n = 18/46) of dogs scored Fair-Poor in the overall fitness score. Sixty-four percent of the dogs sterilised were dehydrated, 26% were underweight, 45% had elevated temperature classified as hyperthermia (i.e. temperature showed steady decreases following repeated measurements). Additionally, 58.7% of dogs were infested with ectoparasites, including ticks and fleas.

A two-way ANOVA was conducted to examine the effects of transport type (walk, car, motorcycle) and ambient temperature (24°C, 26°C) on dogs’ rectal temperature. The interaction between transport type and ambient temperature was not statistically significant (*P* = 0.875), indicating that the effect of transport type on rectal temperature did not differ across the two temperature conditions.

After removing the interaction term, the main effects were examined. Transport type did not significantly affect body temperature (*P* = 0.220). Furthermore, ambient temperature also did not show an effect (*P* = 0.080).

A simple linear regression was performed to examine the relationship between travel distance (km) and dogs’ rectal temperature (°C). Travel distance did not significantly predict rectal temperature (β = 0.007; *P* = 0.770), indicating no detectable change in rectal temperature with increasing travel distance.

### Anaesthesia protocol

The following anaesthetic protocol was used at the clinics; premedication with xylazine (Xyla®) injectable administered intramuscularly, ketamine hydrochloride (Ketamax 50®) injectable as an induction agent and propofol (Nirfol®) for maintenance both administered intravenously.

### Post-surgical assessment

Post-operatively, each dog received a single subcutaneous injection of procaine penicillin & dihydrostreptomycin injection (Penstrep-400®, Interchemie®) to prevent post-operative infections and a single intramuscular injection of meloxicam (Meloxicash®) for pain management.

**Figure 7.**
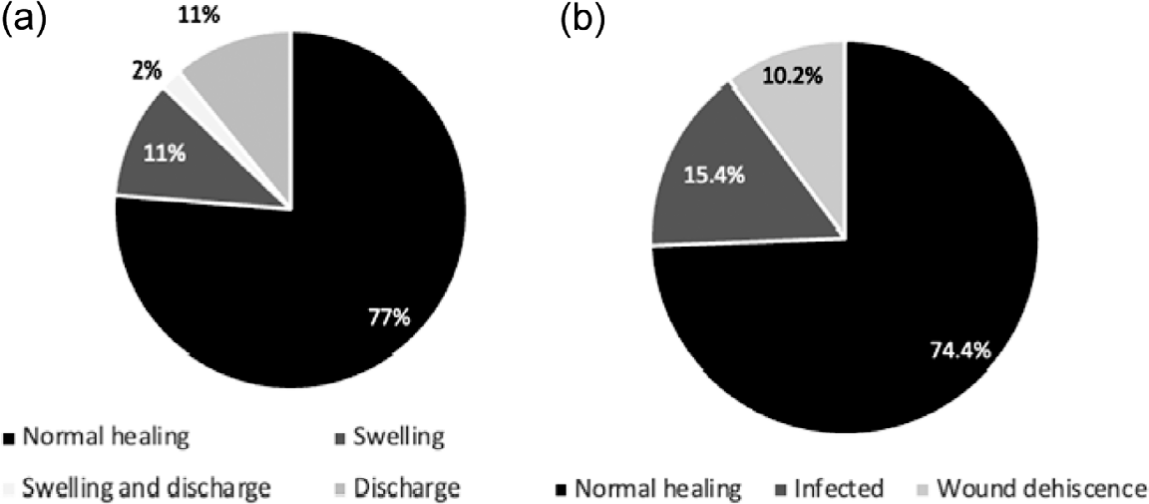
The condition of the incision site of dogs following neutering surgery at the free community pet clinics in Kampala Metropolitan area, Central Uganda on (a) Day 0 (n = 46) and (b) Day 7 (n = 39).

At the clinic, immediately after surgery, 11% of dogs experienced swelling at the site of incision and the same percentage showed both swelling and discharge (Figure 7). Only 2% exhibited only discharge at the incision site.

Seven days later, 84% of participants were visited with the remaining 16% having dropped out of the study due mostly to not being reachable one way or another. It was observed that 13% of dogs who had no complications at Day 0 developed complications (wound dehiscence or infection). Further analysis revealed that 11% of the dogs showing discharge or swelling at Day 0 developed wound dehiscence or infection on Day 7.

The association between overall fitness scores (Excellent-Good vs Fair-Poor) and infection status (normal healing, infection, wound dehiscence) was assessed using Fisher’s Exact test. The results showed no significant association (Fisher’s Exact; *P* = 0.869), indicating that overall fitness scores was not related to infection or wound dehiscence.

### Behavioural change assessment 30 days post-surgery

Some owners dropped out of the study while others were unreachable. We therefore obtained behavioural change data from only 28 of the initial 46 respondents. Of these 28, 13 owners (46.4%) reported having observed an increase in aggressive behaviour in their dog, while the remainder reported no change.

We performed an association analysis between dog behaviour (aggressive vs no change in behaviour) and healing outcomes (normal healing, infection, wound dehiscence) using Fisher’s Exact test. The results indicated no significant association (Fisher’s Exact; *P* = 0.276), suggesting that behaviour change was not related to infection or wound dehiscence in this sample.

## Discussion

We conducted a study to evaluate the welfare of dogs undergoing sterilisation surgery during single-day FCPCs organised in Kampala metropolitan area to identify major gaps in the welfare, and to suggest improvements.

### Mode of transporting dogs to the site

Three modes of dog transportation were reported in this study, i.e. walking the dog and transport either by motorcycle or by car; with walking the most common. The choice of transportation appeared to be influenced by the distance to the clinic. Those living within 2.5 km of the FCPC station generally walked, while owners who lived further than 2.5 km used either private cars or hired public motorcycle transport. Interestingly, those who used public motorcycles often covered the greatest distances.

Some individuals sat on the motorcycle and carried the dog on the lap which is probably comfortable for the dog. However, tying a dog to a motorcycle carrier or packing it in a sack over long distances poses significant risks. These methods can cause discomfort, fear, and potential injury to the dog, all of which represent risk factors that provoke aggression and bites (Overall & Love 2001). A more practical solution might be to advocate for the use of dog carriers temporarily attached to motorcycles. This could improve the comfort and safety of transportation, especially since motorcycles are one of the most affordable and widely available means of transportation in such remote settings (Divall *et al.* 2021; Marija *et al.* 2022).

Although transport by car is generally considered safer for long distances, it is not without its challenges. Studies have shown the potential for car travel to be extremely stressful for dogs, e.g. Beerda *et al.* (1997) found that car transportation can elevate a dog’s salivary cortisol levels by 10 times, indicating severe stress. While Radisavljević *et al.* (2017) concluded that the processes involved in car transportation — such as loading, caging, vehicle vibrations, traffic noise, and unloading — are more stressful for dogs. Furthermore, studies have shown that temperatures inside cars can reach even up to 50°C (Mansor *et al.* 2014), even on cooler days (Grundstein *et al.* 2009). However, in our study, we found no association between transport type and dogs’ core body temperature. Nonetheless, taking into account distances involved and potential for dogs’ post-surgery pain and fatigue, out of the three modes of transport in Uganda, cars undoubtedly represent the safest option. Unfortunately, such an option remains unopen to many owners in remote areas, where cars are often unaffordable (King *et al.* 2022).

### Welfare of dogs at the FCPCs

According to the AWAG, procedural events like handling during assessment, disruptions to daily routines, handling during assessment and procedural pain were the primary contributors to negative welfare states in dogs (3.52), followed by psychological (2.39) and physical factors (1.71). This underscores the fact that certain interventions, in this case surgical sterilisation, can cause stress, fear and discomfort to the dogs involved. This may be due to the invasiveness of the procedure requiring restraint of the dog, which may cause fear and stress. Furthermore, removal from the familiarity of the home environment and being placed in a new location with unfamiliar dogs can exacerbate stress (Lee *et* *al.* 2023). We must mention that procedural pain was not explicitly assessed in this study. Rather, pain scores were assigned according to the descriptor that best reflected the nature of the surgical procedure, as indicated by the AWAG (Malkani *et al.* 2024).While this approach limited the precision of our pain assessment, it nonetheless provided a useful indication of the potential severity of the procedures performed.

Psychological factors, such as aggression towards caregivers and unfamiliar people, as well as reaction to stressors, achieved an AWAG score of 2.39. This implied that most dogs were generally well socialised towards humans and when exposed to a stressor, return to normal within a few minutes.

Physical factors, such as mobility, body condition and clinical assessment scored 1.71. The dogs generally showed good mobility with no lameness and tended to be active. Most dogs were clinically healthy with no injury or sign of disease except for some outliers. However, the majority had poor body condition score with clearly visible ribs and minimal fat covering.

### Provisions

Most of the dogs received no provisions from the clinic organisers, despite spending an average waiting period of 151.4 min at the clinic, prior to surgery, surrounded by and interacting with several unfamiliar animals and humans. In this context, provisions such as drinking water, gentle petting from owners, or an area for play or comfortable rest would be crucial in helping mitigate the inevitable stress caused by a new environment and interacting with unfamiliar dogs (Bacon *et al.* 2017). This might improve both the experience and the recovery process for the dog (Bacon *et al.* 2017).

### Assessment of surgical fitness

A substantial number of dogs (39.0%) were deemed unfit for surgery due to a variety of factors, including poor hydration, low body condition scores, and elevated rectal temperature. The elevated temperatures indicated hyperthermia which likely stemmed from a lack of cooling measures, such as access to shade or drinking water upon arrival at the clinics. Hyperthermia often causes hypotension through peripheral vasodilation, tachycardia, and impaired oxygen delivery (Bruchim *et al.* 2017), all of which compromise anaesthetic safety and tissue perfusion during surgery thereby increasing the risk of intra-operative complications. Although our analysis revealed no statistically significant relationship between transport type, travel distance, or ambient temperature and rectal temperature, this lack of association may have been due to the small sample size.

A considerable proportion of the dogs presented with ectoparasites, in particular fleas and ticks, a factor generally associated with poor animal welfare since heavy infestations are often linked to increased stress and irregular haematological parameters (Kubesy *et* *al.* 2020; Audu *et* *al.* 2022). Such parasitic burdens likely reflect inadequate routine care and limited access to veterinary services in underserved communities (Hyeroba *et al.* 2017; Tayebwa *et al.* 2024b). For many of these dogs, the veterinary care provided during this event may have been the first they had ever received.

A number of dogs were deemed unsuitable candidates for surgery as a result of their poor state of health, however this was insufficient to deter the owners who viewed the FCPC as a rare opportunity to have their animals sterilised and insisted surgery took place. While this may appear cruel from an animal welfare perspective, the owners’ limitations, the scarcity of FCPC events and the perceived benefits for dog-population control all require to be factored in to any decision. In the context of spay-neuter programmes aimed at controlling stray dog populations, Looney *et al.* (2008) and Totton *et al.* (2011) argue that, on balance, the benefits of surgery most likely outweigh the risks.

Some factors are particularly critical for post-surgery recovery. For instance, dogs that are dehydrated are at a greater risk of responding badly to the anaesthesia (Mazzaferro & Wagner 2001). Dehydration can exacerbate the hypotensive effects of general anaesthesia, increasing the risk of surgical complications (Mazzaferro & Wagner 2001). In these cases, excluding dogs from surgery would be an appropriate course of action although this is unlikely to be greeted favourably by owners keen to take advantage of a very rare opportunity. To address this, we recommend that veterinary surgeons implement and communicate a strict pre-evaluation protocol. Any unfit dogs should either be excluded entirely from surgery or be afforded special dispensation through administration of supportive care, for example, isotonic fluids to correct dehydration, thereby preventing fatal outcomes due to unfitness for surgery (Fossum *et al.* 2018; Herstad & Cuq 2023).

### Post-surgical evaluation

A significant number of dogs (25.6%) experienced post-surgical complications when visited one week later. Severe complications, such as wound dehiscence, have been documented as causing fatalities, especially in spayed dogs (Baltodano *et* *al.* 2013), however, there is a paucity of literature regarding infection rates at FCPCs. These post-surgical complications may have arisen as a result of a number of dogs displaying poor health markers at the time of surgery however our analyses show no significant relationships between surgical fitness and outcome. However, several other unexamined intra-operative factors, such as aseptic technique, surgical technique, anaesthetic protocol, as well as post-operative care, inter-sex differences and geographical location could have also contributed to these post-surgical complications. Similarly, effects of transport mode and provisions during pre- and post-surgical periods on likelihood of post-surgical complications were beyond the scope of this study and would be interesting considerations for future research. It is essential to emphasise to the dog owners the importance of closely monitoring the incision site; and for the event organisers to follow-up with the respective owners.

Notably, some of the post-surgical complications suffered by the dogs, consisted of dehiscence and infection. Given the nature of the community clinics, i.e. conducted in outdoor-field settings with limited follow-up, it is perhaps surprising that complications were seen in only 25.6% of dogs. However, this outcome emphasises the importance of enhancing aseptic practice as well as seeking to establish follow-up protocols with owners to ensure adequate and timely post-operative care. On the downside, these additional measures may have implications for cost and require greater time and resources from organisers.

Single-dose, post-operative antibiotics and anti-inflammatory drugs were administered post-surgery. This prophylactic use of antimicrobials post-surgery is controversial due to the risk of antimicrobial-resistant bacteria (Cavalli *et* *al.* 2025). However, Looney *et al.* (2008) noted that when the risk of infection or the potential for complications is high, the use of antimicrobials should be considered acceptable. Since the study surgeries were performed in an outdoor field setting, the use of antimicrobials was deemed acceptable. However, despite the use of antimicrobials, 15.4% of the dogs developed wound infections suggesting that single-dose treatments may be insufficient.

### Behavioural changes

Forty-six percent of owners reported that their dogs displayed increased aggression toward unfamiliar dogs and people in the 30 days following surgery, findings in direct contrast with the commonly held view of castration typically reducing aggressive behaviour in dogs (Hopkins *et al.* 1976; Maarschalkerweerd *et al.* 1997; Neilson *et al.* 1997; Palestrini *et al.* 2021; Kriese *et al.* 2022). However, details regarding the severity and context of such aggressive interactions is unknown and may be attributed to pain experienced following the FCPCs.

Post-operative inflammation and pain typically peak at 24–72 h, beginning to subside by days 3–5, and potentially persisting for 1–2 weeks depending on the procedure performed (Wagner *et* *al.* 2008). Anti-inflammatories, such as meloxicam, are typically administered for 3–5 days post-surgery in dogs, with extension to 7 days indicated for orthopaedic or high-trauma procedures, provided renal function and hydration are monitored (Fuertes-Recuero *et* *al.* 2024). However, in this study, dogs only received one dose of meloxicam at the community clinics with no follow-up doses forthcoming, suggesting they might have experienced pain in the days after the initial dose waned off. Dogs are intelligent and known to internalise adverse experiences, a trait which can influence their future behaviour (Foyer *et* *al.* 2014). Despite this, a conclusion cannot be fully drawn since pain was not assessed in this study. Furthermore, we acknowledge the short time-frame and potential reporters’ bias involved with our behavioural changes assessment. However, it is our assertion that the owners provided a more objective perspective than the assessors since owners spend more time with their dogs allowing observation of subtle changes in behaviour. Future studies investigating behaviour changes following FCPC clinics could help improve the overall experience as well as the safety and welfare of dogs attending such clinics and the community as a whole.

### Study limitations

Our small sample size was a major limitation. This might have reduced the statistical power of analyses, making it more difficult to detect subtle but meaningful differences or associations in transportation, pre-surgical findings, welfare parameters, surgical outcomes, or post-operative behaviour. The small sample size was due to the lengthy nature of the assessment, requiring each selected dog to be followed from the time it arrived at the clinic until departure and even later to assess behavioural change.

Procedural pain was not directly measured in this study. Instead, pain scores were inferred from descriptors within the AWAG framework, based on expected severity and duration of pain associated with surgical procedures. Consequently, the precision of infection-related welfare assessments may have been limited.

Hydration status was primarily assessed using skin turgor, which is influenced by factors such as age, body condition, and skin elasticity. This method may be unreliable in very young, geriatric, or emaciated dogs, potentially under- or overestimating dehydration levels. More comprehensive assessments, such as mucous membrane moisture, capillary refill time, or eye position, would have provided a more accurate evaluation of hydration and surgical fitness but would have also involved prolonged animal handling, potentially increasing stress.

Although post-surgical complications were documented, many contributing factors, such as surgical technique, aseptic protocol and anaesthetic management were not examined. Follow-up was limited to a single visit, one-week post-surgery which may have missed later complications. These factors collectively limit the interpretability and generalisability of post-surgical outcome data.

Behavioural changes, including aggression, were assessed based upon owner reports rather than direct observation or standardised behavioural testing. While owners are familiar with their dogs’ baseline behaviour, reporting bias and the short post-operative observation window may have affected the reliability of these findings. The inability to directly measure pain or fear responses further complicates interpretation of behavioural changes.

### Animal welfare implications

The welfare assessment of dogs presented at the FCPCs revealed multiple concerns regarding animal well-being. Many dogs were underweight, inadequately hydrated, and showing elevated temperatures rendering them poor surgical candidates. However, despite this, they were still operated upon. We encourage the FCPC organisers to only consider healthy dogs for sterilisation surgery to reduce the occurrence of post-operative complications. We identified higher than expected post-surgical complications when dogs were observed several days following the FCPC clinic. We encourage FCPC organisers to include post-surgical monitoring performed by veterinarians, particularly pain assessment following training in order to reduce the long-term welfare issues caused by unmitigated post-operative pain. Additionally, mitigating post-operative pain by providing owners with oral anti-inflammatory options to be administered at home would aid in preventing long-term effects. Transportation practices, particularly post-surgery, often failed to prioritise the animals’ well-being, for example, walking the dogs home while they were still recovering from the effects of anaesthesia, or tying them onto a motorcycle, which risked wound dehiscence and stress. To enhance welfare, targeted owner education, stricter surgical protocols, improved facilities, and follow-up care are essential.

## Conclusion

This study is the first of its kind to provide insights into the welfare of dogs sterilised during FCPCs in Uganda. While FCPCs clearly offer essential veterinary care to underserved communities, benefiting numerous pets (dogs and cats) into the process, several welfare challenges were identified throughout the service chain. These issues include low provision of supporting resources (water, shade, bedding and blankets) at the clinic site, ethical trade-off regarding sterilisation of dogs deemed unfit for surgery, extended waiting periods, and high incidence of post-surgical complications. Even under resource constraints, straightforward measures such as providing drinking water and shaded resting areas, ensuring proper surgical and aseptic technique can improve outcomes. Incorporating these basic practices aligns with veterinarians’ professional responsibility to protect and promote animal welfare, ensuring that population control interventions achieve their intended benefits without causing unnecessary harm. Future FCPCs should prioritise these minimal-cost welfare measures to uphold ethical standards while continuing to address the critical need for sterilisation services. Further studies are recommended to explore the behavioural effects of these events on animals in greater depth.

## Supporting information

10.1017/awf.2026.10071.sm001 Hoareau et al. supplementary material Hoareau et al. supplementary material

## Supplementary material

The supplementary material for this article can be found at http://doi.org/10.1017/awf.2026.10071.
