# Supplementary material for: Assessing the welfare of dogs surgically sterilized during single day free community pet clinics in Kampala Metropolitan area, Central Uganda
Source: Anim Welf. 2026 Feb 18;35:e14. doi: 10.1017/awf.2026.10071 (PMC12926845; doi:10.1017/awf.2026.10071)
Supplement: Hoareau et al. supplementary material [file S0962728626100712sup001.pdf]

Assessing the welfare of dogs surgically sterilised during single day free community pet clinics in Kampala Metropolitan area, Central Uganda: Supplementary material

Zozianne Keenan Hoareau-Kakoozahttps://orcid.org/0009-0006-6478-044X[1], Lewis Ashabahebwahttps://orcid.org/0009-0008-1742-8405[1], William Lume[2], Kelvin Bwambale[3], Suzanne T Millman[4], Dickson Stuart Tayebwahttps://orcid.org/0000-0002-3116-7538[1,5]

[1] Department of Veterinary Pharmacy and Clinical Studies, College of Veterinary Medicine Animal Resources and Biosecurity, Makerere University, PO Box 7062, Kampala, Uganda

[2] Director of Jinja Institute of Technology, PO Box 1078, Jinja, Uganda

[3] Department of Biostatistics and Epidemiology College of Health Sciences, Makerere University, PO Box 7062, Kampala, Uganda

[4] Department of Veterinary Diagnostic and Production Animal Medicine, Iowa State University, 1809 South Riverside Drive Ames, Iowa 50011, USA

[5] Department of Animal Health, Vetconekt Initiative, Kampala, Uganda

Author for correspondence: Dickson Stuart Tayebwa, email: tayebwa.dickson@gmail.com

Section 1

Table S1. Animal Welfare Assessment Grid showing welfare parameters (physical, psychological, procedural), their factors (e.g., for physical parameter; mobility, body condition and clinical assessment), and the descriptors for each factor score (1=lowest impact on welfare, 10=highest impact on welfare) (Malkani *et al.* 2022) with modifications.

Physical		
Mobility	Body Condition	Clinical Assessment
1 The dog has very good mobility with no lameness and is normally active or has normal energy	1 Ribs easily palpable without pressure, with minimal fat covering, waist easily noted and evident abdominal tuck	1 Clinically healthy, no injury or sign of disease
2 Very good mobility with occasional mild stiffness and is normally active	2 Ribs fairly easy to palpate without pressure, with thin fat covering, and evident abdominal tuck from above	2 mild transient subclinical symptoms or injury but has no evident behavior change or impact on welfare
3 good mobility with short boats of stiffness	3 Slight fat covering, slight pressure needed to palpate ribs, waist observable from above	3 Mild transient clinical symptoms or injury with mild transient behavior change and impact on welfare

4 good mobility with generalized of stiffness	4 slight covering of fat, slight waist observable from above, can palpate ribs with pressure needed	4 mild clinical symptoms or injury with mild behavior change and impact on welfare
5 moderate mobility, stiffness but frequently active	5 moderate covering of fat, waist discerned from above but not prominent, can palpate ribs with pressure	5 moderate transient clinical symptoms or injury with some behavior change and impact on welfare
6 moderate mobility, stiffness and less active	6 excess covering of fat, no discernable waistline and difficulty palpating ribs	6 moderate clinical symptoms or injury with moderate behavior change and impact on welfare
7 poor mobility, stiffness and less active	7 (overweight) heavy fat present and slight abdominal distention, difficult to palpate ribs or (underweight) ribs and shoulder visible with little fat	7 moderate/severe disease or injury with moderate behavior change and impact on welfare
8 very poor mobility, stiffness and less active	8 (overweight) heavy fat present with abdominal distention, cannot palpate ribs or (underweight) ribs, lumbar and vertebrae and pelvic bones somewhat	8 moderate/severe disease or injury with severe behavior change and impact on welfare

	visible with little detectable fat	
9 very poor mobility, stiffness and not active at all	9 (Overweight) very heavy fat present with obvious abdominal distention, cannot palpate ribs or (underweight) ribs, lumbar vertebrae and pelvic bones easily visible with little fat	9 severe disease and clinical symptoms or injury with severe behavior change and impact on welfare
10 non-ambulatory and cannot move without assistance or support		10 Extreme disease with clinical symptoms or injury with extreme behavior change and impact on welfare
Psychological		
Aggression towards caregiver	Aggression toward unfamiliar people	Reaction to stressors
1 none	1 none	1 displays minimal signs of fear and anxiety when encounters potential stressors
2 occasionally growls, is predictable and trigger avoided	2 occasionally growls, is predictable and trigger avoided	2 shows signs of fear to stressors and returns to normal <30s

3 occasionally growls, is predictable but trigger not always avoided	3 occasionally growls, is predictable but trigger not always avoided	3 shows signs of fear to stressors and returns to normal in minutes
4 occasionally growls, is predictable but trigger rarely avoided	4 occasionally growls, is predictable but trigger rarely avoided	4 shows signs of fear to stressors and some minor and returns to normal after 10 minutes
5 occasionally snaps or bites, is predictable and trigger avoided	5 occasionally snaps or bites, is predictable and trigger avoided	5 shows signs of fear to stressors returns to normal after 30 minutes
6 occasionally snaps or bites, is predictable but trigger not always avoided	6 occasionally snaps or bites, is predictable but trigger not always avoided	6 shows signs of fear to stressors and takes up to an hour to return to normal
7 occasionally snaps or bites is predictable, but trigger rarely avoided	7 occasionally snaps or bites is predictable, but trigger rarely avoided	7 shows signs of fear to stressors and takes several hours to return to normal
8 bites, is somewhat predictable and trigger largely avoided	8 bites, is somewhat predictable and trigger largely avoided	8 shows signs of fear to stressors and takes most of the day to return to normal
9 bites, is somewhat predictable and trigger not avoided	9 bites, is somewhat predictable and trigger not avoided	9 shows signs of fear to stressors and takes

		several days to return to normal
10 severe bits that are unpredictable	10 severe bits that are unpredictable	10 shows signs of fear to stressors and is always anxious

Procedural			
Behavior during assessment	Change in daily routine	Handling during assessment	Procedure pain
1 is calm and actively seeks interaction from assessor/s	1 Procedure/disruption to day <15 minutes	1 displays minimal signs of stress when handled, is calm and tolerates being handled will	1 no procedure required
2 is mostly relaxed and shows mild signs of stress to few triggering events	2 Procedure/disruption to day <30 minutes	2 minimal movement when handled, sometimes licks lips, yawns or shows other appeasement behavior	2 minor procedure with no expected pain
3 is somewhat relaxed and shows mild signs of stress to some triggering events	3 Procedure/disruption to day 30 minutes to1 hour	3 minimal movement handled, licks lips, yawns or shows appeasement behavior frequently	3 minor procedure longer duration with no expected pain

4 is not relaxed and shows moderate signs of stress to few triggering events	4 Procedure/disruption to day 1-2 hours	4 some slow movement when handled, turns head away from handle, slow panting, displays more than two signs of stress such as ears back and tail down	4 minor procedure with short mild pain
5 shows moderate signs of stress to some triggering events	5 Procedure/disruption to day 3-4 hours	5 moderate movement when handled, fast panting, displays more than two signs of stress such as ears back, tail tucked and furrowed brow	5 moderate procedure with short duration of transient pain
6 shows moderate signs of stress to all triggering events	6 Procedure/disruption to day >4 hours	6 some attempt to escape, fast movements, tense body and tense closed mouth	6 moderate procedure, longer in duration with transient pain
7 shows major signs of stress to	7 Procedure/disruption to day >6 hours	7 moderate attempts to escape, fast movements or frozen	7 moderate/severe procedure with pain lasting >12 hours

few triggering events		and staring, tense and trembling	
8 shows major signs of stress to some triggering events	8 Procedure/disruption to day >8 hours	8 strong attempts to escape when handled or frozen, lifts lips and shows teeth	8 severe procedure with pain lasting >24 hours
9 shows major signs of stress to all triggering events	9 Procedure/disruption to day >12 hours	9 will violently attempt to escape when handled or frozen, growls and barks	9 severe procedure with pain or complications lasting >48 hours
10 cannot cope being in the environment, is extremely shut down or aggressive and shows major signs of stress	10 Procedure/disruption to day >24 hours	10 cannot be handled, growls and attempts to bite when approached.	10 extensive procedures resulting in severe long-term pain or complications

Section 2

RAVS Animal Condition Score (RACS)

The RACS is intended to provide an assessment of the overall general physical condition of an individual dog as a reflection of the level of care provided.

The assessment considers the health and welfare of the animal because of client-controlled factors.

The primary measure is a simple five-point subjective assessment scale:

RAVS Animal Condition Score: Excellent Good Fair Poor Critical

Only health factors that can be directly influenced by the client are considered. The focus is on the CURRENT physical condition of the animal. Medical history is not considered as the goal is to evaluate the animal's present condition.

RACS[1] - SCORING DESCRIPTIONS

Excellent: Animal appears to have all health, and welfare needs met and good general level of care.

- o Good body condition (BCS 4-5/9).
- No indication of parasite infestation.
- Temperature within normal range.
- Adequate hydration

Good: Essential health/ welfare needs met and appropriate general level of care.

---

[1] Rural Animal Veterinary Services

- Minor acute illness/injury or chronic problem that has been diagnosed/under veterinary care.
- May be slightly under/over-weight (BCS 3/9 or 6-7/9).
- Parasite infestation (not significantly impacting quality of life.)
- Temperature within normal range
- Adequate/Marginal hydration

Fair: Basic needs being met, but little additional care provided.

- Moderate illness/injury that may impact quality of life or lifespan if left untreated.
- May be underweight (BCS 3/9)
- Parasitic infestation or mange.
- Temperature within normal range
- Marginal hydration

Poor: Some care provided, but basic needs not being adequately met.

- Significantly underweight (BCS < 3/9) or grossly obese (BCS 8-9/9).
- Parasite infestation or mange with possible secondary infections.
- Temperature outside normal range (hypothermic or hyperthermic)
- Inadequate hydration

Critical: Seriously neglected, no attention to basic health/husbandry needs.

- Nutritional status completely inadequate (BCS 1-2/9).
- Severe parasite infection / severe generalized mange with secondary infection.
- Temperature outside normal range (hypothermic or hyperthermic)

- Inadequate hydration
- Likely to die without immediate veterinary attention and/or immediate change in level of care.
